# Malignant Tumors of the Mouth

**Published:** 1895-12

**Authors:** 


ARTICLE II.

### MALIGNANT TUMORS OF THE MOUTH.

During the operation a portion of the root was broken off and allowed to remain. From this time the parts were sensitive and easily irritated, and a few weeks later a small tumor developed which was probably cystic or soon became so, for, he says, it was opened many times by phy sicians and dentists, always discharging a thin water-like fluid, but never discharging any pus.

Six years ago it began to increase in size, but made slow progress till about two months ago, when a change came on rapidly, and at the time he entered the University Hospital, March 14th, 1895, the growth had extended forward on the lower jaw, nearly to the median line, backward to the angle and well up the ramus, pushing the tissues of the cheek prominently outward. There was an opening in the central portion of the tumor, from which flowed a thin offensive fluid; the patient stated that some small pieces of bone had been discharged through the opening, but no trace had been discovered of the offending root. All the molar teeth on that side have recently been extracted, and their place is now occupied by the growth.

A probe inserted in the opening revealed the fact that some fragments of bone still remained, which would undoubtedly come away soon if the tumor is allowed to pursue its course unmolested.

Excepting these, a large portion of the bone seems to be destroyed or displaced by the growth,

Excision of the right half of the lower jaw, together with the outlying parts of the growth, offered the best chance for a cure, and the patient promptly selected this course.

The operation did not present any unusual points of difficulty, except that the bone was easily broken at the point of disease, making it much more difficult to remove the ramus.

Recovery has promptly taken place, and at this time he is well. In this case is found, what was at first a very simple tumor, a dentigerous cyst or epulic growth, after many years of irritation changing to a sarcoma. The dread which the physician, dentist or patient may have of operation, or their faith in medical measures, probably were responsible for the delay which had allowed this change to take place. This case is an earnest appeal to early and complete removal of all tumors of the mouth, except in those cases where the growth is not irritated, remains stationary, and can be frequently seen by the surgeon or dentist in charge, and operation advised, when the slightest change for the worse takes place.

Sarcomatous tumors of the jaw may be removed with a reasonable hope of cure when the growth is central (myeloid sarcoma), if the entire bone to which the disease is confined be removed. Periosteal sarcoma does not promise such good results, because the surrounding tissue may be involved, but these tumors come early in life when the patient is vigorous and will rapidly recover from operations. In all tumors of this class, not only the jaw should be removed to the median line, but the entire periosteum and attachments of muscles must also be taken away, if we operate with any expectation of a cure. Another tumor of the mouth, remarkable because of its slow growth and change, occured in a patient who entered the University Hospital in June, 1894, during my term of service in the clinic.

Mrs. D., sixty-six years old, was for many years troubled with catarrh. Twelve years ago she first noticed a growth in the hard palate to the left of the median line. She was wearing a plate at the time and believed that it may have irritated the mouth and thus caused a tumor to develop, at least the irritation was soon so great that she could not wear it.

The tumor probably began in the hard palate or the floor of the left antrum; was a very slow growth till a few months since, when it began to rapidly increase in size. It involved all the border of the left maxilla, nearly all of the hard palate and much of the soft palate. The left antrum and the nasal passages were filled by the extension of the growth upward. It projected so far downward that the mouth could scarcely be closed. An exploring needle passed in the tumor did not come in contact with the bone where the border of the maxilla and the hard palate should be found. A portion of the tumor was excised for microscopical examination and the pathologist reported carcinoma.

The malignant nature of the tumor and its certain fatal result if allowed to remain, together with the danger of a return of the disease if the tumor was removed, were all explained to the patient. She expressed a strong desire to have the operation made, and it was done, Professor Nancrede kindly assisting me. Tracheotomy was first performed, and the larynx packed with a sponge, that we might continue the anæsthetic and the operation at the same time, without having blood enter the trachea, as it certainly would with the severe hemorrhage we must encounter unless such means were taken to prevent it. An incision was carried from the middle of the lip upward to the left of the nose and under the left eye, the flap was then reflected to give sufficient room for cutting the bone. The alveolar portion was first divided in the median line, then the molar and nasal portions, after which the diseased part was torn from its resting place by the lion forceps. The alveolar border was cut away at least one inch to the right of the median line, and all the surface from which the growth was removed was scraped with a curette to remove any small particles of diseased tissue that might be remaining. Hemorrhage was stopped by means of the Paquelin cautery, and the cavity was packed for forty eight hours with iodoform gauze.

The patient made a good recovery, and returned to her home in a few weeks, free from pain and relieved of the presence of such a tumor. When last heard from she was entirely well. The disease in this case may return, but not for some months. During this time she will be free from pain, and death may result from some other disease.

Here is another case illustrating the proper method of operating for carcenoma when the surgeon sees his case early.

Mr. W. H., farmer, has been a moderate drinker, enjoying good health. Three weeks ago he first noticed a small growth on the under surface of the right side of his tongue; it was closely connected with the surrounding tissue, growing rapidly and painlessly. A small portion was excised under cocain anæsthesia, and submitted to microscopical examination. October 17th, 1894, he was operated on in professor Nancrede's clinic, one half of the tongue being removed well back to the base. Rather a heroic operation for a small growth of three weeks' duration you may say, but here is the pathologist's report: "I have made sections of the growth from the tongue already reported as epithelioma, and as far as I can see you have gone well outside of the disease." This extensive operation was the only hope of cure for this man, even then the lymphatic glands may have been involved, and the return of the disease may be noted at that point, while the scar surface remains healthy. Cancer appearing in the mouth can scarcely be mistaken for any other growth, and there is never any difficulty in removing a portion to confirm the diagnosis, then prompt and thorough removal is the only chance for cure. When any lymphatics are found to be suspiciously enlarged, they must also be removed at the same time.

Mechanical devices to take the place of parts removed or to correct deformities following operations for malignant disease should not be applied for some time after complete recovery, because of the danger that such irritation might renew the disease. When the cicatrices are well formed or the diseased condition is not malignant nor beyond the bone, this point may be disregarded and the deformity corrected. The use of medicine in the treatment of malignant tumors is not to be considered where operation is possible but may be tried as a last resort. Coley, of New York, has recently reported thirty-five cases treated by injecting the combined toxines of erysipelas and the bacillus prodigiosus. In five of these cases he has reasonable hope of a permanent cure. All of his cases were inoperable and the diagnosis was verified microscopically. The investigations being carried on at the present time concerning the causes and treatment of these tumors certainly promise great results.

While we wait for these promises to become truths, let early and complete operation be the leading treatment of all tumors of the mouth, then few of our patients can say:

> "Oh, that comfort comes too late,
> 'Tis like a pardon after execution,
> That gentle physic given in time had cured me
> But now I am past all comfort here but prayers."

*Dental Register.*

